# Genome-wide identification and characterization of TIFY family genes in Moso Bamboo (*Phyllostachys edulis*) and expression profiling analysis under dehydration and cold stresses

**DOI:** 10.7717/peerj.2620

**Published:** 2016-10-27

**Authors:** Zhuo Huang, Si-Han Jin, Han-Du Guo, Xiao-Juan Zhong, Jiao He, Xi Li, Ming-Yan Jiang, Xiao-Fang Yu, Hai Long, Ming-Dong Ma, Qi-Bing Chen

**Affiliations:** 1 College of Landscape Architecture, Sichuan Agricultural University, Wenjiang, Sichuan, China; 2 Chengdu Institute of Biology, Chinese Academy of Sciences, Chengdu, Sichuan, China

**Keywords:** TIFY protein, Dehydration, Gene family, Cold stress, Transcriptome sequencing, Moso bammboo (*Phyllostachys edulis*)

## Abstract

The proteins containing the TIFY domain belong to a plant-specific family of putative transcription factors and could be divided into four subfamilies: ZML, TIFY, PPD and JAZ. They not only function as key regulators of jasmonate hormonal response, but are also involved in responding to abiotic stress. In this study, we identified 24 TIFY genes (*PeTIFYs*) in Moso bamboo (*Phyllostachys edulis*) of Poaceae by analyzing the whole genome sequence. One *PeTIFY* belongs to TIFY subfamily, 18 and five belong to JAZ and ZML subfamilies, respectively. Two equivocal gene models were re-predicted and a putative retrotransposition event was found in a ZML protein. The distribution and conservation of domain or motif, and gene structure were also analyzed. Phylogenetic analysis with TIFY proteins of *Arabidopsis* and* Oryza sativa* indicated that JAZ subfamily could be further divided to four groups. Evolutionary analysis revealed intragenomic duplication and orthologous relationship between *P. edulis*, *O. sativa*, and *B. distachyon*. Calculation of the non-synonymous (Ka) and synonymous (Ks) substitution rates and their ratios indicated that the duplication of *PeTIFY* may have occurred around 16.7 million years ago (MYA), the divergence time of TIFY family among the *P. edulis*-*O. sativa*, *P. edulis*-*B. distachyon,* and* O. sativa-B. distachyon* was approximately 39 MYA, 39 MYA, and 45 MYA, respectively. They appear to have undergone extensive purifying selection during evolution. Transcriptome sequencing revealed that more than 50% of *PeTIFY* genes could be up-regulated by cold and dehydration stresses, and some *PeTIFYs* also share homology to know TIFYs involved in abiotic stress tolerance. Our results made insights into TIFY family of Moso bamboo, an economically important non-timber forest resource, and provided candidates for further identification of genes involved in regulating responses to abiotic stress.

## Introduction

The TIFY proteins, containing a conserved motif TIF[F/Y]XG in the TIFY domain, constitute a plant-specific family of putative transcription factors (Vanholme et al., 2007). The first TIFY gene identified was originally known as ZIM (Zinc-finger protein expressed in Inflorescence Meristem, *At4g24470*), which was highly expressed in reproductive tissue, and described as a putative transcription factor (Nishii et al., 2000). Based on structural and phylogenetic analyses, TIFY proteins could be divided into four groups, including (ZIM)/ZIM-like (ZML), TIFY, PEAPOD (PPD) and jasmonate-ZIM-domain (JAZ) subfamilies (Vanholme et al., 2007; Bai et al., 2011). Excepting for TIFY subfamily exclusively containing the TIFY domain, the ZML proteins contain a C2C2-GATA zinc-finger DNA-binding domain and a CCT domain (CONSTANS, CO-like, TOC1), the JAZ subfamily contains a C-terminal conserved sequence, designated Jas motif, which is similar in sequence to the N-terminal portion of the CCT domain with a characteristic SLX2FX2KRX2RX5PY motif (Chung et al., 2009; Staswick, 2008), the PPD proteins bear a unique N-terminal PPD domain, as well as a divergent Jas motif (Chung et al., 2009).

Some TIFY proteins have been functionally characterized. The expression of *AtTIFY1/AtZIM* was detected in all tissues (Nishii et al., 2000). Its over-expression resulted in petiole and hypocotyl elongation, which was independent of gibberellin and brassinosteroids (Shikata et al., 2004). Function loss of *AtTIFY4a/AtPPD1* and *AtTIFY4b/AtPPD2* caused multiple morphological alterations on leaf shape, silique length, and stomata number (White, 2006). In *Arabidopsis*, the JAZ subfamily has been intensively investigated as key regulators of jasmonate hormonal response. They repress responses to jasmonate via interacting with basic helix-loop-helix (bHLH) transcription factors, which regulate the expression of downstream genes (Bai et al., 2011; Chini et al., 2007; Melotto et al., 2008; Thines et al., 2007; Yan et al., 2007). AtTIFY10b/JAZ1 plays important role in JA signal transduction. It could be degraded by JA through the SCF^COI1^-dependent 26S proteasome pathway to remove the repression of JA-response genes (Thines et al., 2007). OsTIFY3/OsJAZ1 is involved in spikelet development through interacting with OsCOI1b and OsMYC2 (Cai et al., 2014). Another rice JAZ gene, *OsTIFY11b/OsJAZ10* could increase grain-size by enhancing accumulation of carbohydrates in the stem (Hakata et al., 2012).

TIFY genes are also involved in abiotic stress responses (Bai et al., 2011; Chung et al., 2008; Ye et al., 2009). Over-expression of *OsTIFY11a* could enhance tolerance to salt and dehydration stresses in rice (Ye et al., 2009); *OsTIFY11a* and *OsTIFY11c* are involved in salt tolerance (Toda et al., 2013), and *OsTIFY11d* transcriptionally regulate the OsbHLH148-mediated JA signaling pathway leading to drought tolerance in rice (Ye et al., 2009). *AtTIFY10a10b* and their homolog in wild soybean, *GsTIFY10a*, are positive regulators of plant alkaline stress (Zhu et al., 2014). These results indicate that TIFY family might be valuable resource for stress responsive genes.

The rapidly accumulation of plant whole genome sequences provides opportunities to genome-widely identify and characterize the genes encoding TIFY proteins in plant, which will not only provide insights into their evolution process, but also offer basis for further identification of candidate genes regulating stress responses. Such work has been performed in several genome-sequenced plant species, such as *Arabidopsis* (Vanholme et al., 2007), *Oryza sativa* (Ye et al., 2009), grape (Zhang et al., 2012), *Phaseolus vulgaris* (Aparicio-Fabre et al., 2013), *Glycine soja* (Zhu et al., 2013), apple (Li et al., 2015), *Gossypium raimondii* (He et al., 2015) and *Brachypodium distachyon* (Zhang, You & Chan, 2015), etc.

Bamboo, comprising of more than 1,400 species, belongs to Bambusoideae of Poaceae. Most components of Bambusoideae are perennial and arborescent species, which grow large woody culms up to 30 cm in diameter and 12 m in height (Barker et al., 2001). Fast growing, high productivity, strongly regeneration capability make it become to one of the most important non-timber forest resources in the world. Investigation of stress-responsive genes in bamboo will facilitate genetic improvement of stress tolerance to cope with the increasing environmental challenges. However, little is known about its responses to abiotic stress and underlying mechanism at molecular level. This might be partly due to the lack of genomic resources.

Recently, the genome of Moso bamboo (*Phyllostachys edulis*), a large woody bamboo with high ecological and economic values, were decoded (Peng et al., 2013). In this study, we searched the *P. edulis* genome to identify the genes encoding TIFY proteins (PeTIFYs). Classification, characterization and phylogenetic analyses were conducted. Duplication, orthologous relationship and timing of divergence among the representatives of three subfamilies of Poaceae (the BEP clade), Bambusoideae (*P. edulis*), Ehrhartoideae (*O. sativa*) and Pooideae (*B. distachyon*), were analyzed. Dehydration and cold induced expression profiling was also performed to identify abiotic stress induced *PeTIFY* genes.

## Materials and Methods

### Data resources

The whole genome dataset, full length cDNA, and expressed sequence tags (ESTs) of *P. edulis* were downloaded from the bamboo genome database (www.bamboogdb.org/) or National Center for Biotechnology Information (www.ncbi.nlm.nih.gov/). These include 31,987 protein-coding genes predicted from whole genome sequences, 10,608 low redundant full-length cDNA sequences and 38,000 ESTs from leaf, shoot, and seedling libraries. The deduced amino acid sequences of TIFY genes in *A. thaliana* genome and rice genome (*O. sativa subsp. indica)* were according to Vanholme et al. (2007) and Ye et al. (2009), respectively. The corresponding nucleotide sequences of coding region (cds) and genomic sequences were obtained from The Arabidopsis Information Resource (TAIR, www.arabidopsis.org) and *O. sativa* genome database (rice.plantbiology.msu.edu).

### Identification of TIFY genes

To identify all possible members of the TIFY gene family in Moso bamboo, the *Arabidopsis* TIFY sequences were first submitted to the Pfam database (http://pfam.sanger.ac.uk) (Finn et al., 2010) to obtain the domain architecture of this family. The TIFY domain, Jas and CCT motifs were represented by Pfam accession numbers PF06200, PF09425 and PF06203, respectively. The corresponding profiles were downloaded and used to search the Moso bamboo Genome Database (www.bamboogdb.org/) by using HMMER (Eddy, 1998). The obtained candidates were further confirmed by the Pfam database. The full length cDNA and EST datasets, comprising of 10,608 low redundant full-length cDNA sequences and approximately 38,000 ESTs from leaf, shoot, and seedling libraries, were employed to obtain evidences for the obtained genes.

### Multiple alignments and phylogenetic analysis

The amino acid sequences of TIFY genes were aligned by using MUSCLE (Edgar, 2004) with default parameters, and the phylogenetic tree was then constructed by using MEGA5.2 software with Neighbor-Joining method (Tamura et al., 2011). The Jones-Taylor-Thornton (JTT) model and a discrete Gamma distribution (+G) with 5 rate categories were chosen based on model test. The bootstrap test was carried out with 1,000 iterations.

### Analyses of exon-intron structures and protein motifs

The coordinates of exon and intron of TIFY genes of *P. edulis* were extracted from their corresponding scaffolds and exon-intron structures were illustrated using Gene Structure Display Server (GSDS, http://gsds.cbi.pku.edu.cn/) (Guo et al., 2007). The consensus amino acid sequences of TIFY domain and Jas motif were illustrated by using WebLogo (Crooks et al., 2004).

### Evolutionary analyses of the paralogs and orthogos in *P. edulis, O. sativa* and *Brachypodium*

Reciprocal BLASTP was carried out to establish orthologous relationship among *P. edulis*, *O. sativa* and *Brachypodium*. The hits threshold values were set as E-value <1e–10, score >200, and positive >70%. The paralogous relationship within *P. edulis* was also analyzed with more stringent parameters of E-value <1e–50, score >200, and positive >80%. To further confirm the paralogous and orthologous relationship, the nucleotide sequences of coding sequences were also aligned and phylogenetic tree was constructed by using p-distance model. The bootstrap test was carried out with 1,000 iterations.

The synonymous (Ks) and non-synonymous (Ka) substitution rates of paralogous and orthologous were analyzed by Ka_Ks calculator 2.0 (Zhang et al., 2006). Time (million years ago, Mya) of duplication and divergence was calculated using a synonymous mutation rate of one substitutions per synonymous site per year as T = Ks/2*λ* (*λ* = 6.5 × 10^−9^) (Lynch & Conery, 2000; Yang et al., 2008).

### Stress treatment and RNA-seq

To evaluate expression patterns of *PeTIFY* genes under abiotic stress, dehydration and cold treatments were conducted. The *P. edulis* plants used in this study were manually planted and grown under natural condition at Linyanshan Experimental Base (N31°0′33.20″, E103°36′51.95″) of Sichuan Agricultural University, Dujiangyan, Sichuan, China. The approximately 20 cM branch containing full young unexpanded leaves of 4–5 centimeters long were cut from five two-year old *P. edulis* plants in similar growth status. All these samples were collected in the morning at 10, October, 2015 (approximately at 10 o’clock and it was cloudy with temperature of ∼22 °C). For dehydration treatment, the branches were placed on the dry filter paper and treated under room temperature (20 °C and 50% humidity). For cold treatment, the branches were put into a chamber set to 0 °C without light. At 2h and 8h after each treatment, ten individual unexpanded leaves were detached from base and immediately frozen in liquid nitrogen, from which the total RNA was extracted according to the manual of the TRIZOL RNA Kit (TIANGEN, Beijing, China). The same amount of untreated leaves were also sampled and immediately frozen in liquid nitrogen, which were used as control. The qualities and quantities of extracted nucleotide were measured by NanoDrop 2000 Spectrophotometer (Thermo Fisher, USA) and Agilent 2100RNA 6000 Nano kit.

The cDNA library construction and sequencing on Illumina HiSeq™ 4000 platform were performed by Onmath Co. (Chengdu, China), following the manufacturer’s standard protocol. The sequences of 150 bp in length were generated as raw data by pair-end sequencing strategy. The filtered clean reads were mapped to all obtained *PeTIFY* genes by using TopHat v2.0.9. HTSeq v0.6.1 was used to count the reads numbers mapped to each gene. And then RPKM of each gene was calculated based on the length of the gene and reads count mapped to this gene. RPKM, Reads Per Kilobase of exon model per Million mapped reads, considers the effect of sequencing depth and gene length for the reads count at the same time, and is currently the most commonly used method for estimating gene expression levels (Mortazavi et al., 2008).

### Quantitative real-time PCR (qPCR)

The cDNA was synthesized with Oligo18dT primer by using single-stranded cDNA Synthesis Kit (TaKaRa, Dalian, China) following the manufacture introduction. qPCR reactions were conducted employing an BioRad CFX system using the iQ SYBR Green supermix kit (BioRad). The PCR procedure was: pre-incubation at 95 °C for 5 min followed by 40 cycles of denaturation at 95 °C for 15 s, annealing at 60 °C for 15 s, and extension at 72 °C for 15 s. The *elongation factor 1a* (*EF1*) (Long et al., 2010; Liang et al., 2012) was used as internal control to quantify the relative transcript level. The primers with a single peak on the melting curve were chosen for qPCR analyses (Table S1). The target and internal control were amplified in separate wells in triplicate. The Cq values were determined automatically by BioRad CFX manager 2.1 (BioRad) and the mean Cq of triplicate was used to calculate the relative level of gene expression by using the 2^−ΔΔ*ct*^ method (Livaka & Schmittgen, 2001). The samples from three independent biological replicates were analyzed and compared to those obtained by RNA-seq.

## Results and Discussions

### Identification of TIFY genes in *P. edulis*

To identify putative TIFY proteins from *P. edulis* genome, the hidden Markov model (HMM) profiles of the TIFY domain, Jas and CCT motifs were downloaded from Pfam database and utilized to screen the *P. edulis* genome data by using HMMER. A total of 24 proteins containing a TIFY domain were identified, in which five proteins contain both a TIFY domain and a CCT motif, 17 proteins contain both a TIFY domain and a Jas motif (also known as CCT_2 motif), respectively. Two proteins, PH01000549G0400 and PH01000361G0580, only contain a TIFY domain. The domain or motif of these proteins were re-analyzed by Pfam and showed consistent results with those of HMMER. Additionally, a C2C2-GATA zinc-finger domain (GATA) was found in three out of five proteins containing both a TIFY domain and a CCT motif (Table 1). Using these protein sequences as queries, we searched the EST and full length cDNA of *P. edulis*. As a result, 16 TIFY genes obtained evidence from expressed sequences (Table 1).

**Table 1:** Information of *TIFY* family in *P. edulis*.

Gene ID	Subfamily	Length (amino acids)	TIFY motif	Evidence	identity	e-value
PH01000008G2960	JAZ	427	TIFYDG	bphyem207g19	100	0
PH01000038G0470	JAZ	208	TIFYGG	bphyem107e01	100	e–116
PH01000038G0510	JAZ	173	TIFYGG	bphyem202h03	82	7.00E–77
PH01000052G0540	JAZ	272	TIFYGG	JG297588.1		
PH01000114G0660	ZML	303	TLSFQG			
PH01000115G0020	JAZ	205	TIFYGG	bphyem118f19	100	e–115
PH01000115G0040	JAZ	183	TIFYGG	bphyem202h03	100	7.00E–99
PH01000158G0210	JAZ	235	TIFYGG			
PH01000213G1380	JAZ	164	TIFYDG	bphyem116j13	81	2.00E–70
				JG297216.1		1.00E–92
PH01000213G1410	JAZ	178	TIIYGG	bphylf050a14	81	2.00E–73
PH01000310G0500	JAZ	245	TIFYGG	bphyem118e13	100	e–135
PH01000360G1030	JAZ	242	TIFYGG	bphyem205j14	100	e–136
PH01000361G0580	JAZ	209	TIFYDG			
PH01000549G0400	TIFY	504	IIFYDG			
PH01000597G0660	JAZ	208	TIFYGG	bphyem115l15	93	e–103
PH01000750G0690	ZML	258	TLVYQG	GH205920.1		1.00E–83
PH01000836G0660	ZML	470	TLSFQG			
PH01000878G0620	ZML	1718	TLSFQG			
PH01001078G0280	JAZ	163	TIFYGG			
PH01001078G0420	JAZ	190	TIFYDG	bphyem116j13	99	e–104
				GH205920.1		2.00E–81
PH01001584G0350	ZML	344	TLLFQG			
PH01001852G0020	JAZ	183	TIFYGG	bphyem122g05	100	e–100
PH01002950G0020	JAZ	411	TIFYAG	JG296527.1		e–125
PH01144128G0010	JAZ	193	TIFYDG	bphylf025d08	100	e–103

Interestingly, two GATA domains are present in PH01000114G0660, whereas no GATA domains was found in one protein PH01000878G0620, who also has both the TIFY domain and CCT motif. We also noticed that PH01000361G0580 contains a incomplete TIFY domain with a rather lower E-value (9.70E–05). Therefore, to further validate these equivocal gene models, we retrieved their corresponding scaffolds and re-predicted the gene models by GENESCAN (Burge & Karlin, 1998). As results, PH01000114G0660 was predicted to be same as previously presented. Two novel gene models were harvested: PH01000361G0580 was re-predicted to encode a protein of 209 amino acids rather than previous 83 amino acids, containing both the TIFY domain and Jas motif (Table 1, Table S2, and Fig. S1). PH01000878G0620 was predicted to have a coding sequence (CDS) of 5,157 bp, which encodes a protein of 1718 amino acids, containing a TIFY domain, a Jas motif, as well as a GATA domain. The length of genomic sequence of the new PH01000878G0620 is 22,593 bp, which is much longer than previous 2,253 bp (Tables 1, S2, and Fig. S1). Comparing to two homologous proteins in *O. sativa* and *Brachypodium* (LOC_Os06g48534.1 and Bradi1g33980), a peptide comprising of 676 amino acids seems to be inserted between CCT motif and GATA domain of PH01000878G0620, and additionally, it is fused with another protein of 794 amino acids at C-terminal domain (Fig. S2). Protein annotation by Pfam and DELTA-BLAST algorithm on NCBI website indicated that the 676 amino acids insertion consists of an UBN2_3 domain (gag-polypeptide of LTR copia-type), a RVT_2 (reverse transcriptase) peptide, and a RNase_HI_RT_Ty1 (Ty1/Copia family of RNase HI in long-term repeat retroelements) peptide (Table S3, Fig. S2). The C-terminal of PH01000878G0620 could be annotated as Arsenite-resistance protein 2 (ARS2), and comprises a DUF3546 (Domain of unknown function) domain (Table S2 and Fig. S2). These results indicate that a putative retrotransposition event may have occurred leading to putative structural variation of PH01000878G0620.

### Classification and gene structure

**Figure 1:**
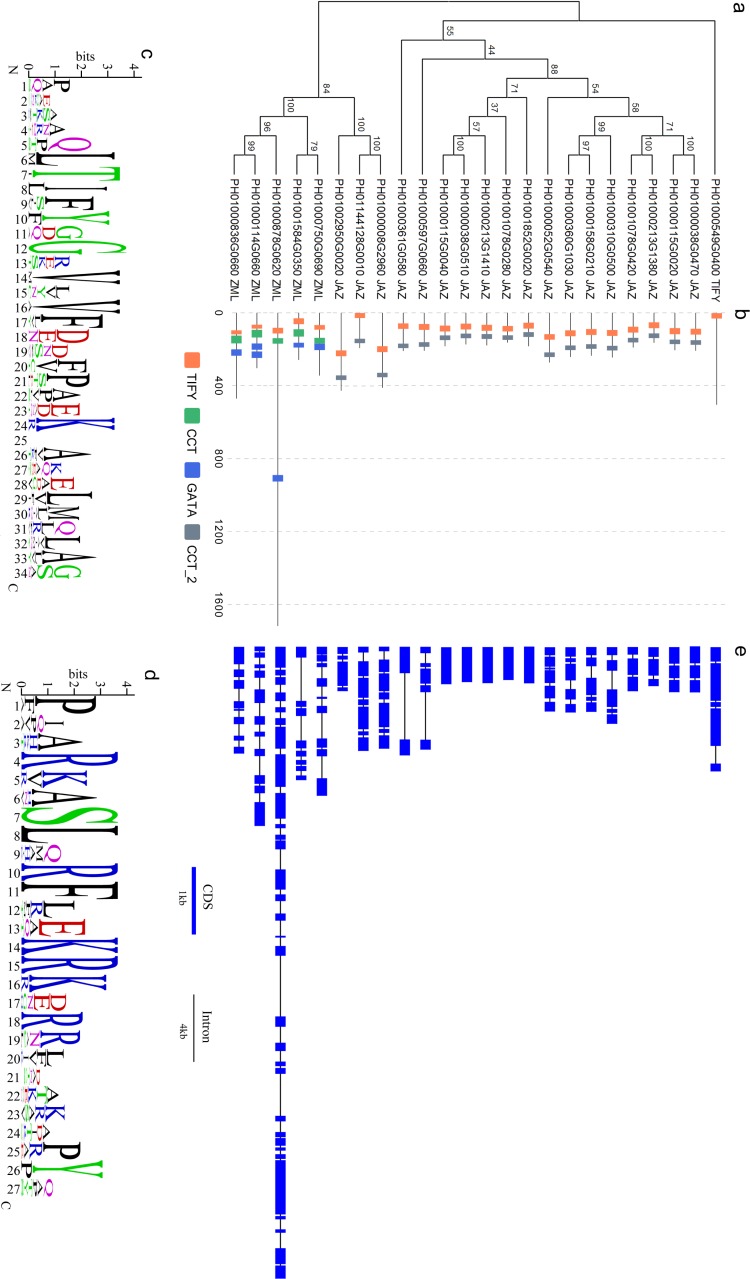
Sequence analyses of PeTIFY proteins. (A), Phylogenetic tress of PeTIFYs based on the alignment of deduced amino acid sequences; (B), Distribution of characteristic domains and motifs; (C) and (D), Consensus sequences of TIFY domain and Jas motif present by motif logo; (E), exon-intron structures of *PeTIFY* genes.

Based on the multiple alignment of PeTIFY protein sequences, a neighbor-joining tree was constructed (Fig. 1). It showed that the 18 TIFY proteins containing a TIFY domain and a Jas motif were clustered together and they were assigned as JAZ subfamily; Five proteins containing a TIFY domain a CCT motif, and a GATA domain were clustered closely and belong to ZML subfamily; One protein only contained a TIFY domain and was clustered separated from others, which was assigned to TIFY subfamily. Compared with some plant species (Bai et al., 2011), *P. edulis* shares similar gene member to those of monocots, such as *O. sativa*, *B. distachyon*, and *Sorghum bicolor*, but different from those of dicots species.

By drawing the sequence logos, we analyzed conservation of TIFY domain and Jas motif (Fig. 1C). Besides TIFY motif, Gly-12 (position is according to that present in Fig. 1C), Val-14, Val-16, Phe-17, and Lys-24 are conserved crossing the 24 PeTIFYs. Compared with TIFY domain, the Jas motif has more conserved residues (Fig. 1D). The JAZ proteins play important roles in Jasmonates-mediate responses to biotic and abiotic stresses, as well as some important development progresses (Wasternack, 2007). For example, some JAZ proteins are transcriptional repressors of Jasmonates-responsive transcription factors (such as MYC2 and MYC3). With the accumulation of Jasmonates, JAZ will interact with CORONATINE-INSENSITIVE1 (COI1), and trigger the ubiquitination degradation of JAZ proteins mediated by COI1- E3-type ubiquitin ligase SCF (Skp/Cullin/F-box) complex (SCF^COI1^), and the de-repression of transcription factors (Yan et al., 2007; Thines et al., 2007; Melotto et al., 2008). Due to the essential role in this interaction and Jasmonates sensitivity, Jas motif is highly conserved in both diocts and monocots (Fig. S3).

The exon/intron structures among the 24 TIFY genes of *P. edulis* are divergent. Except for five genes containing an uninterrupted open reading frame, all others showed exon/intron structures. It was also found that some genes with close phylogenetic relationship also share conserved exon/intron structure. We identified four genes pairs, PH01000038G0470/PH01000115G0020, PH01000213G1380/PH01001078G0420, PH01000310G0500/PH01000158G0210, and PH01002950G0020/PH01000008G2960, exhibited same number of exons with highly similar length to each other, respectively (Fig. 1E).

### Phylogenetic analysis of TIFY family among *P. edulis*, *O. sativa*, and *Arabidopsis*

**Figure 2:**
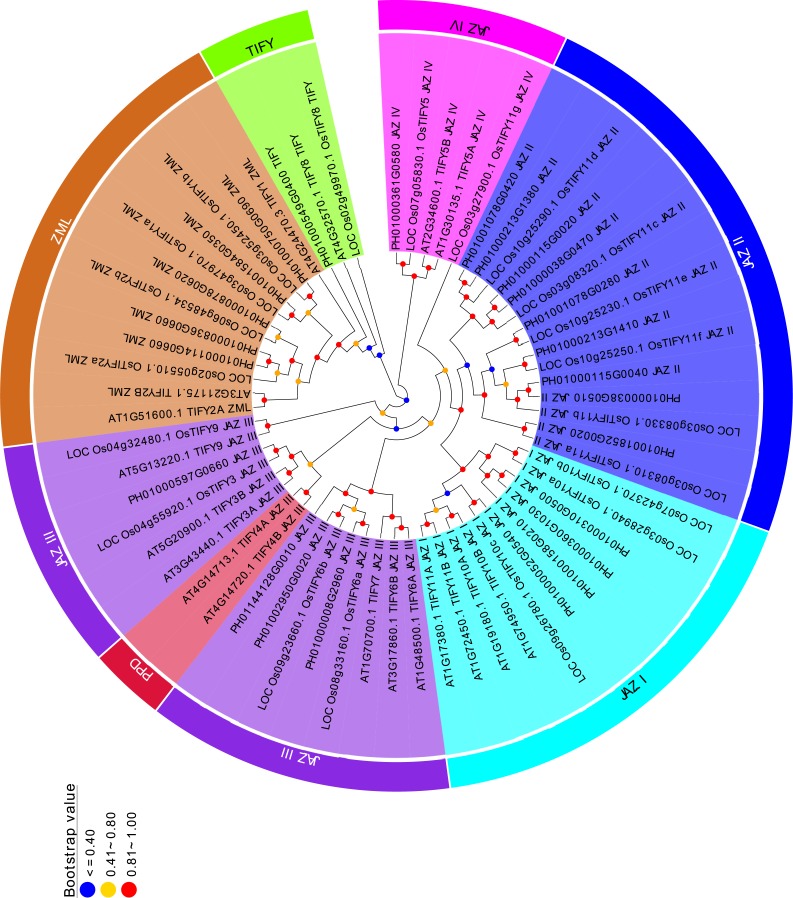
Phylogenetic analysis of PeTIFYs, OsTIFYs, and AtTIFYs. The amino acid sequences of TIFY genes were aligned by using MUSCLE (Edgar, 2004) and the phylogenetic tree was then constructed by using MEGA5.2 software with Neighbor-Joining method (Tamura et al., 2011).

To analyze the evolutionary relationships of the TIFYs, a Neighbor-Joining tree was constructed based on the alignment of 24 PeTIFYs, 20 TIFYs of *O. sativa*, and 18 TIFYs of *Arabidopsis* (Vanholme et al., 2007). According to the phylogenetic tree, all 62 proteins could be separated to several clusters (Fig. 2). All ZML subfamily members were clustered together. PH01000549G0400, LOC_Os02g49970.1 and AT4G32570.1 were grouped closely, which belong to the TIFY subfamily and were close to ZML subfamily. All proteins of JAZ subfamily formed several groups. JAZ I group is composed of four PeTIFYs, three OsTIFYs, and four AtTIFYs, respectively. JAZ II group is close to JAZ I, consisting of nine PeTIFY proteins. No AtTIFY was clustered in this group. Similar results were found in previous studies (Ye et al., 2009; Zhang, You & Chan, 2015), suggesting that group JAZ II might be specific for monocots. Four PeTIFYs were grouped together with eight AtTIFYs and for OsTIFY proteins, which were assigned to group JAZ III. The repredicted PH01000361G0580 was grouped closely with two AtTIFYs and two OsTIFYs, which were assigned as group JAZ IV.

Among group JAZ III, two PEAPOD (PPD) proteins of *Arabidopsis* were cluster separately. The PPD protein, containing an N-terminal PPD domain, was firstly identified by map-based cloning in ppd mutants (White, 2006), in which PPD1 has been suggested to coordinate tissue growth, modulate lamina size, and limit the curvature of the leaf blade (White, 2006), while the PPD2 was found to interact with the geminivirus AL2 protein and the coat protein promoter (Lacatus & Sunter, 2009). They have been found in dicot species, such as *Populus trichocarpa*, *Mimulus guttatus*, *Glycine max*, *Medicago trunculata*, and *Vitis vinifera*, etc (Bai et al., 2011; Zhang et al., 2012), but had not been identified in monocots.

### Duplication, orthologous relationship and divergence rates among *PeTIFYs, OsTIFYs,* and * BdTIFYs*

*P. edulis, O. sativa* and *B. distachyon* are representatives of Bambusoideae, Ehrhartoideae and Pooideae, the three subfamilies of grass (BEP clade), whose phylogenetic relationship was just resolved recently (Wu & Ge, 2012; Peng et al., 2013). It is interesting to investigate the evolutionary relationship of TIFY family between the three species. By reciprocal BLASTP analyses, 15 *OsTIFYs* (75%) and 12 *BdTIFYs* (57%) are found to be orthologous to 20 *PeTIFYs* (83%), respectively (Table S4).

**Figure 3:**
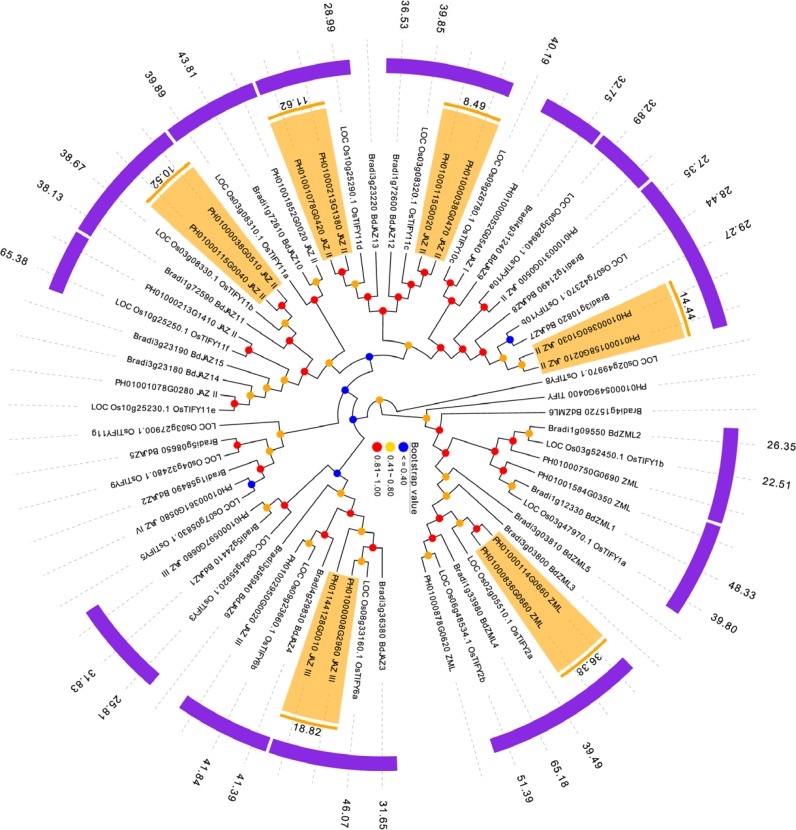
Duplication and orthologous relationship between *P. edulis, O. sativa*, and *B. distachyon*. The duplicated *PeTIFY* genes are highlighted, color stripes indicate orthologues, numerical values indicate estimated divergent time (million years ago).

Whole genome duplications, such as tandem and segmental duplications usually give rise to copy numbers of a gene family and have been considered as important drive force of occurrence of novel biological functions. The reciprocal BLASTP analysis revealed that 12 *PeTIFYs* are involved in six paralgous gene pairs, in which one pair belongs to ZML subfamily, and the other five belong to JAZ subfamily, respectively. We also conducted phylogenetic analyses of three species to support the paralogous and orthologous relationship identified by BLAST analyses. According to the phylogenetic tree, the paralogues or orthologues were clustered together with high bootstrap values (Fig. 3).

**Figure 4:**
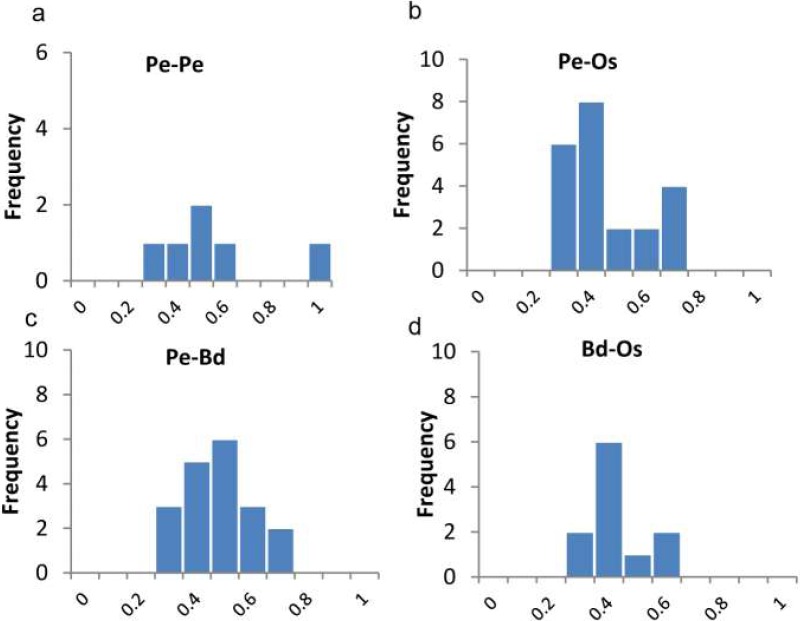
Ratios of non-synonymous (Ka) and synonymous (Ks) substitution rates. as showing in Table S1. Pe-Pe indicates Ka/Ks distributions of paralogues of *P. edulis*, Pe-Os, Pe-Bd, Bd-Os indicate Ka/Ks distributions of *P. edulis-O. sativa*, *P. edulis-B. distachyon*, and *B. distachyon-O. sativa* orthologue pairs.

In order to evaluate the timing of intragenomic gene duplication events, as well as divergence of orthologoues, the synonymous substitution rate (K_*S*_) was calculate. The paralogous gene pairs exhibited mean Ks around 0.22 (Fig. 3). Then estimated by universal substitution rate of 6.5 ×10^−9^ mutations per site per year, the duplications of *PeTIFYs* may have occurred around 16.7 million years ago (MYA) (Fig. 4). Peng et al. (2013) reported a putative recent whole genome duplication at 7-12 MYA based on analyzing whole genome duplication events of *P. edulis*. Wu et al. (2015) found that *P. edulis* genome underwent a large-scale event around 15 MYA by genome-wide analysis of AP2/ERF transcription factors. Our result is similar to these reports and supported that the expansion of *PeTIFYs* occurred along with the genome duplication event. Among the *P. edulis*-*O. sativa*, *P. edulis* –*B. distachyon*, and *O. sativa- B. distachyon* orthologous gene pairs, the mean Ks are approximately 0.51, 0.51, and ∼0.58, indicating that their divergent time was ∼39 MYA, 39 MYA, and 45 MYA, respectively (Fig. 4A). This is consistent with that inferred by whole genome analyses (Peng et al., 2013).

We also calculated the ratios of non-synonymous (Ka) versus synonymous (Ks) substitution rate (Ka/Ks) for duplicated gene-pairs as well as the orthologues of *O. sativa* and *B. distachyon* (Fig. 4B). The Ka/Ks ratio is a measure of the selection pressure to which a gene pair is subjected. Ka/Ks <1 means purifying or negative selection, Ka/Ks = 1 stands for neutral selection, and Ka/Ks >1 indicates positive selection (Lynch & Conery, 2000). The Ka/Ks for paralogous gene pair of *PeTIFYs* are 0.3–0.91 with mean of 0.5. Those for orthologous genes pairs of *PeTIFY-OsTIFY*, *PeTIFY-BdTIFY*, and *OsTIFY*-*BdTIFY* are 0.23–0.68 with mean of ∼0.38, 0.26–0.59 with mean of ∼0.38, respectively (Fig. 4B). These results indicated that TIFY family in the three species appear to have undergone extensive purifying selection during evolution.

### Abiotic stress-induced expression profiling

Plant hormones, such as abscisic acid (ABA), jasmonic acid (JA), salicylic acid (SA), and ethylene (ET) are small signaling molecules involved in stress response. The TIFY family were initially found to be involved in JA signaling pathway. The further genome-wide analyses indicated that most of TIFY genes are regulated by JA and ABA, but not SA and ET (Zhang et al., 2012; Li et al., 2015). As both phytohormones are responsive to biotic and abiotic stresses, the TIFY genes may have versatile roles in stress responses. In *Arabidopsis*, genes of TIFY family, especially JAZ subfamily, are induced by wounding, herbivory, and pathogen infection (Chung et al., 2008; Demianski, Chung & Kunkel, 2012). Additionally, several *AtTIFY* genes in *Arabidopsis* are also found to be upregulated by ozone (Rao et al., 2000), and high salt concentrations (Jiang & Deyholos, 2006). Since bamboo is one of the fast growing and economically important non-timber forest resources in the world, abiotic stress can have enormous negative impact on its production. Therefore, it is meaningful to excavate *PeTIFY* genes responsive to abiotic stress, such as dehydration and cold.

**Figure 5:**
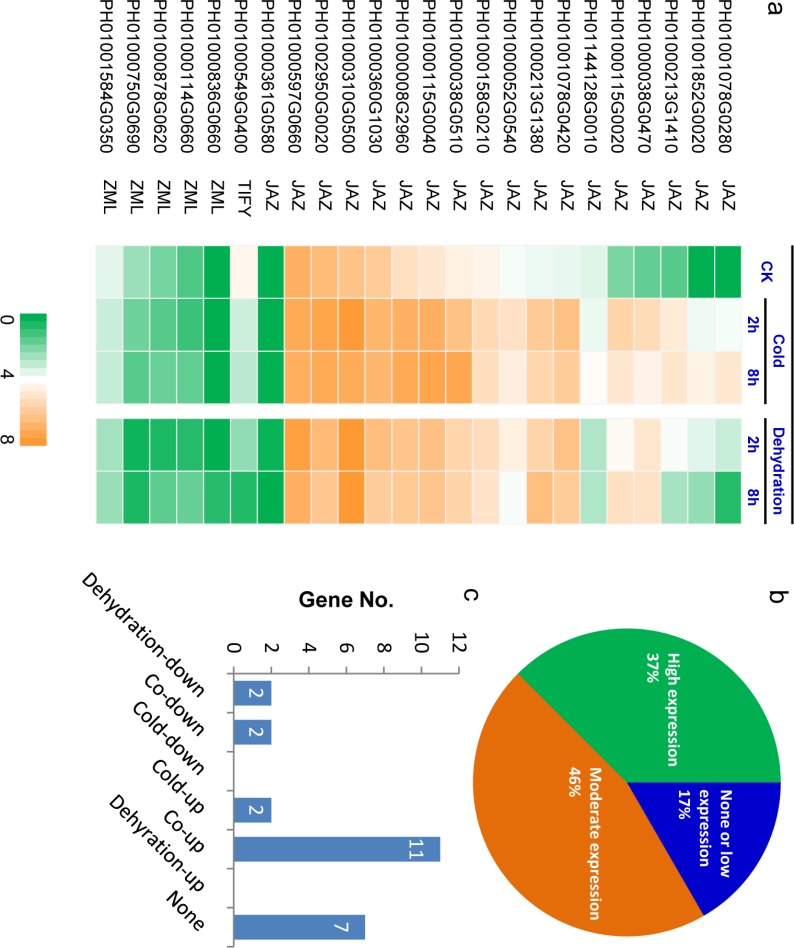
Expression profiling of *PeTIFY* family under dehydration and cold stresses. (A), Heatmap of expression values (showed by log_2_ RPKM values) in control, and in treated samples 2 h and 8 h after treatment; (B) Percentage of genes in different expression levels under normal conditions; (C) Genes numbers exhibiting different responses to dehydration and cold. The expression data is obtained through two biological replicates. The relatedness of two replicates was present in Fig. S4.

We performed transcriptome sequencing to evaluate dynamic expression levels of *PeTIFY* genes under dehydration and cold stresses (Fig. 5A). Approximately four giga bases high quality data (error rate in base calls is less than 0.1%.) for each sample were generated and used to calculate RPKM values of *PeTIFYs.* Two biological replicates were performed and showed high degree of correlations (Fig. S4). We further validated transcripts levels by using qPCR. As results, most of the genes exhibited similar expression patterns to those harvested by RNA-seq (Fig. S5), indicating high reliability of RNA-seq data in this study.

Approximately 37% of *PeTIFYs* are highly expressed (log2 RPKM value > 4) in leaf under normal conditions, 46% showed moderate expression levels (log2 RPKM value between 1 and 4), and the remaining 17% are in very low expression level or not expressed (Figs. 5A and 5B). Among the two repredicted genes, PH01000361G0580 seemed not expressed, whereas PH01000878G0620 showed low expression level. Most of the highly expressed *PeTIFY* genes are of JAZ subfamily, whereas, all ZML subfamily member showed rather low expression level or no expression. As the JAZ proteins are also involved in regulation of some important developmental progresses, such as seed germination, root growth, and flower development (Wasternack, 2007), we proposed that the *JAZ* genes of *P. edulis* expressed under normal conditions may function in development process of *P. edulis*.

Under dehydration and cold stresses, 11 and 13 TIFY genes are up-regulated (RPKM fold change > 2), two genes are down-regulated, while seven genes are not responsive to the two stresses (Figs. 5A and 5C). All *PeTIFY* genes up-regulated by dehydration could also be up-regulated by cold. Similar phenomenon was found in different plant species. In grape, the majority of the TIFY genes were responsive to osmotic and cold stress, almost all of genes exhibiting responses to cold were also regulated by drought or salinity (Zhang et al., 2012). In apple, 10 of the 22 apple TIFY genes respond to drought or high salinity, and among six were upregulated by both treatments (Li et al., 2015). These results suggested that these genes may have multiple functions in responding to the different abiotic stresses.

Five *PeTIFYs*, PH01001078G0280, PH01001852G0020, PH01000213G1410, PH01000038G0470, and PH01000115G0020, show low expression levels under normal condition and are quickly responsive to both dehydration and cold, in which PH01001852G0020, and PH01000038G0470 and PH01000115G0020 are homologous to *OsTIFY11a* and *OsTIFY11c* of rice, respectively. These two genes function in salt and dehydration tolerance (Ye et al., 2009; Toda et al., 2013). Additionally, PH01001078G0420 and PH01000213G1380 are also quickly respond to stress (Fig. 5A). They are homologous to *OsTIFY11d* involved in drought tolerance (Ye et al., 2009). The correspondence between expression patterns and homology to function known TIFY genes suggest that they may have similar functions in stress responses. Further work is necessary to functionally characterize the roles of these stress-induced *PeTIFY* genes in responding to abiotic stress.

## Conclusion

In this study, we identified 24 genes encoding TIFY proteins from *P. edulis* gene model by HMMER. The gene number is similar to those of monocots, but different from those of dicots. The motif distribution and exon/intron structure revealed conservation and divergence among PeTIFYs. The motif composition and a phylogenetic analysis between PeTIFYs, OsTIFYs, and AtTIFYs indicated that 18 PeTIFYs belong to JAZ subfamily, five are assigned to ZML subfamily, and one belongs to TIFY subfamily, no PPD protein was found. The JAZ subfamily could be further divided to four groups. We performed evolutionary analyses among *P. edulis*, *O. sativa* and *B. distachyon*, the representatives of Bambusoideae, Ehrhartoideae and Pooideae subfamilies of grass (BEP clade). Six duplicated paralogues are found in PeTIFYs, whereas 15 *OsTIFYs* and 12 *BdTIFYs* are orthologous to 20 *PeTIFYs*, which are phylogenetically close to each other. The timing of duplication events, as well as divergence of orthologoues were estimated by mean Ks values. Calculation of Ka/Ks indicated that TIFY family in the three species may have undergone extensive purifying selection during evolution. By RNA-seq and qPCR validation, we identified 11 and 13 *PeTIFYs* responsive to dehydration and cold stresses, respectively, in which some of them are homologous to known *OsTIFY* genes functioning in abiotic stress tolerance. These *PeTIFY* genes could be considered as potential resource for genetic improvement of abiotic stress tolerance of *P. edulis* in the future.

##  Supplemental Information

10.7717/peerj.2620/supp-1 Figure S1 Comparison of length and conserved domain or motifs of two PeTIFY proteins before and after re-prediction

10.7717/peerj.2620/supp-2 Figure S2 Comparison of length and conserved domain or motifs of a re-predicted PeTIFY protein with its homologues in *B. distachyon* and *O. sativa*

10.7717/peerj.2620/supp-3 Figure S3 Comparison of motif logos of TIFY domain and Jas motif between different plant species The motif logos of *B. distachyon*, *Gossypium raimondii*, and grape were derived from those reported by Zhang et al. 2015, He et al. 2015, and Zhang et al. 2012.

10.7717/peerj.2620/supp-4 Figure S4 Relatedness of expression level evaluated by two biological replicates

10.7717/peerj.2620/supp-5 Figure S5 Quantitive real-time PCR (qPCR) validation of expression levels of *PeTIFYs* evaluated by RNA-seq X axis: D2, and D8 indicated expression levels at 2-hour and 8-hour under cold treatment (a), and G2 and G8 indicated expression levels at 2-hour and 8-hours under dehydarion treatment (b), respectively. Y axis indicated fold changes of expression levels, in which the expression level of CK was normalized to 1. Each PeTIFY gene was represented by gene ID starting with G as showing in Table S1.

10.7717/peerj.2620/supp-6 Table S1 Primers used for qPCR analyses

10.7717/peerj.2620/supp-7 Table S2 Comparison of Pfam analyses of two re-predicted gene models

10.7717/peerj.2620/supp-8 Table S3 Detailed protein annotation of re-predicted PH01000878G0620 by Pfam and NCBI

10.7717/peerj.2620/supp-9 Table S4 Paralogous and orthologous relationship among PeTIFY, OsTIFY, and, BdTIFY

10.7717/peerj.2620/supp-10 Table S5 Expression levels of PeTIFY genes under dehydration and cold stresses Note: Expression level was present as RPKM value and log2 Normalized.Dehy indicates Dehydration.

## Additional Information and Declarations

### Data Availability

The following information was supplied regarding data availability: The raw data has been supplied as a supplementary file (Table S4).
