# Supplementary figures and images for: Genome-wide identification and characterization of TIFY family genes in Moso Bamboo (Phyllostachys edulis) and expression profiling analysis under dehydration and cold stresses
Source: PeerJ. 2016 Oct 27;4:e2620. doi: 10.7717/peerj.2620 (PMC5088587; doi:10.7717/peerj.2620)

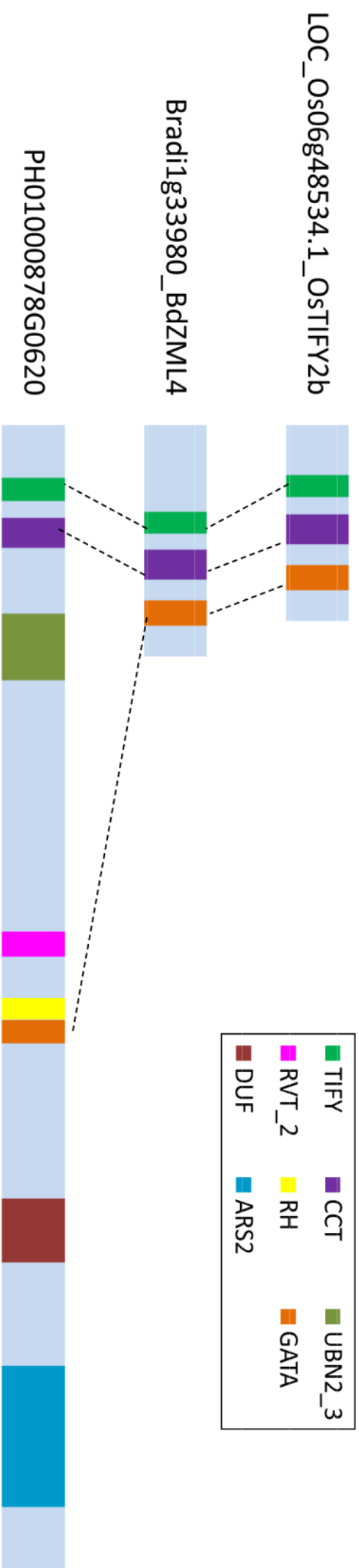

Supplement: Figure S2 [file peerj-04-2620-s002.pdf]

# TIFY domain

Pe

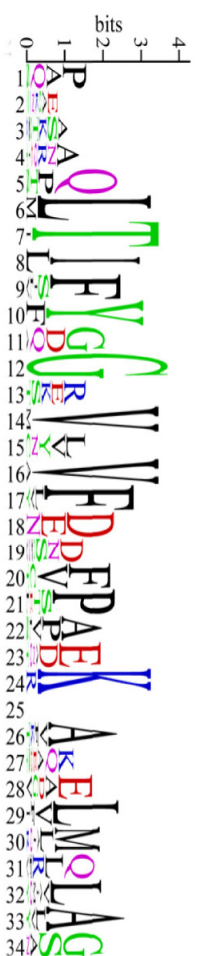

Bd

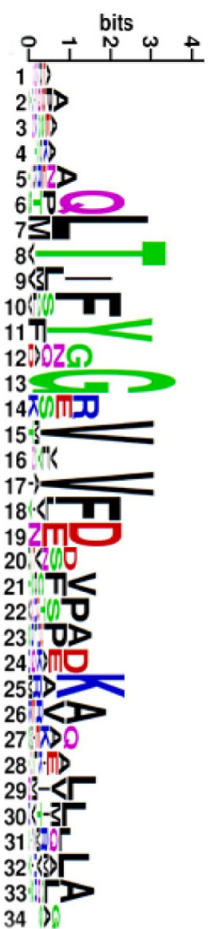

Gr

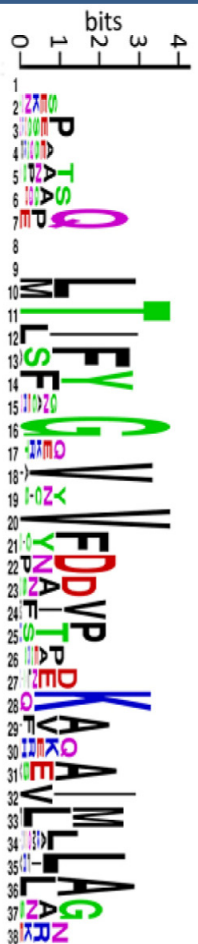

grape

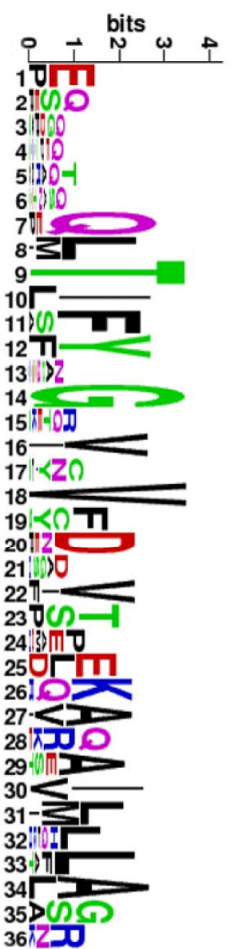

# Jas motif

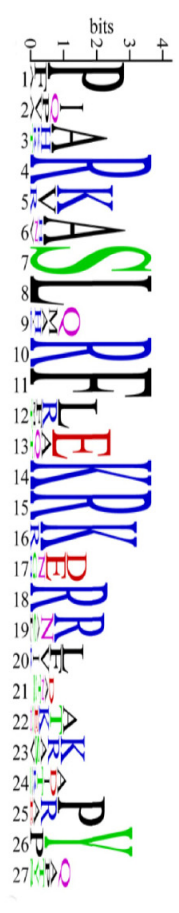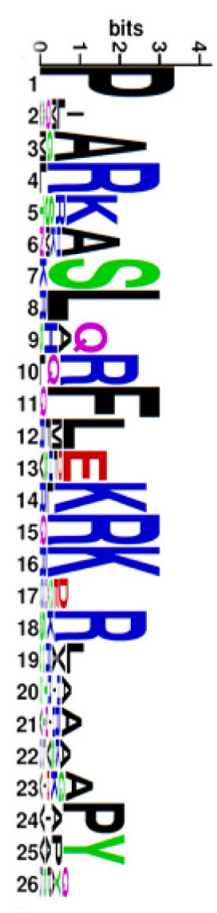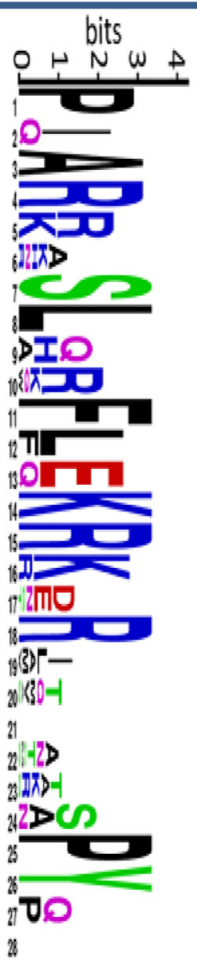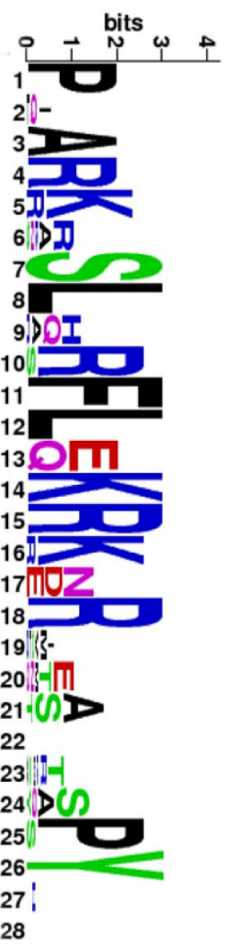

Supplement: Figure S3 — The motif logos of B. distachyon, Gossypium raimondii, and grape were derived from those reported by Zhang et al. 2015, He et al. 2015, and Zhang et al. 2012. [file peerj-04-2620-s003.pdf]

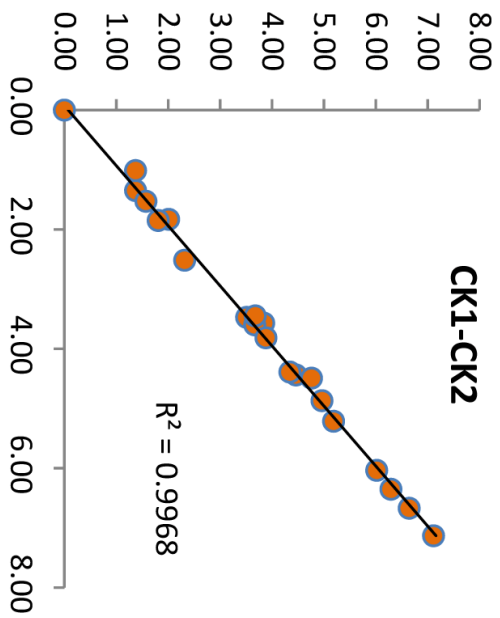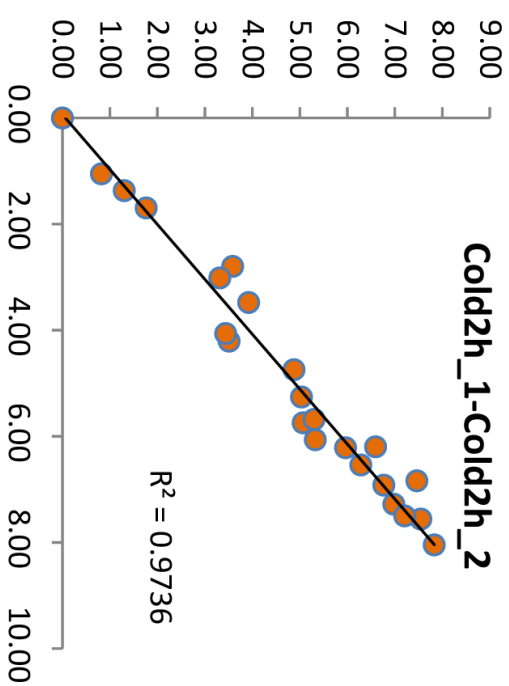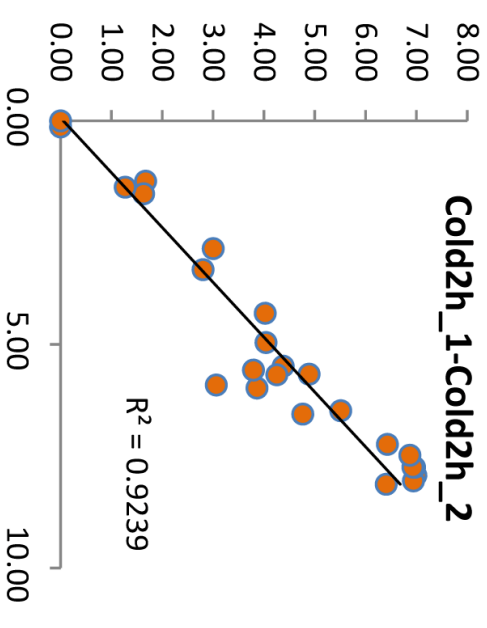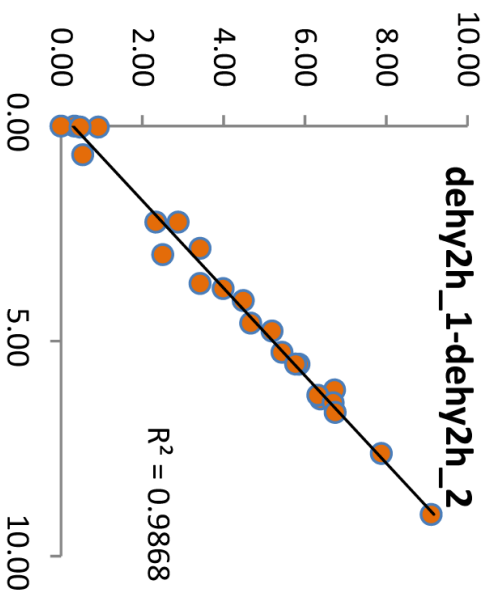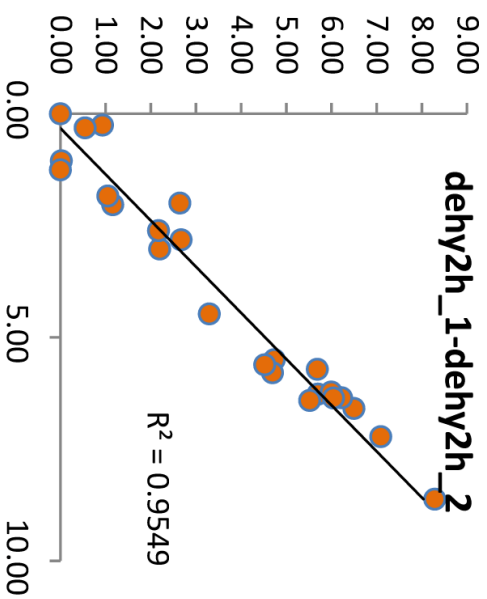

Supplement: Figure S4 [file peerj-04-2620-s004.pdf]

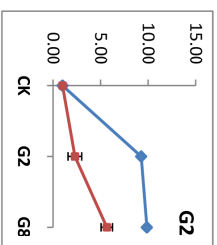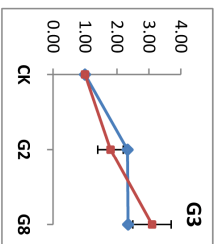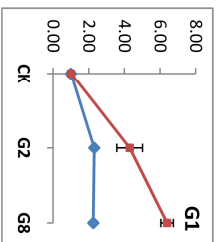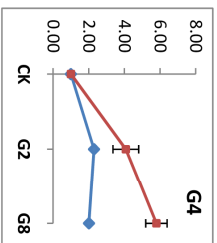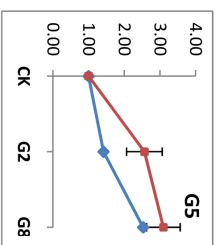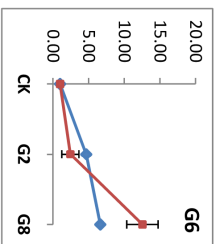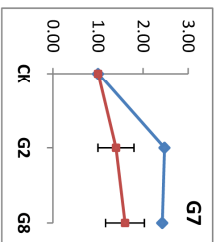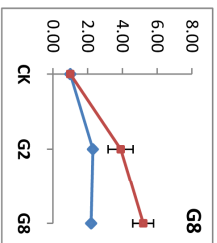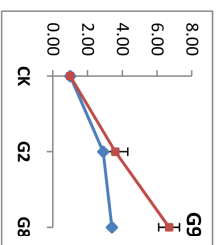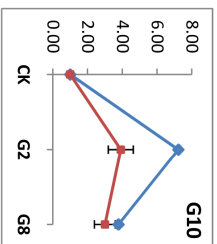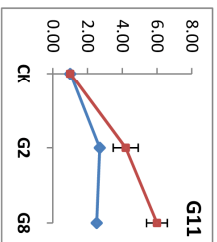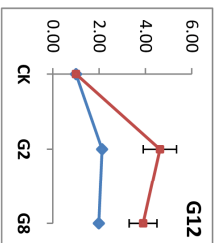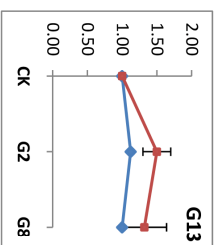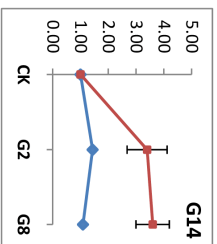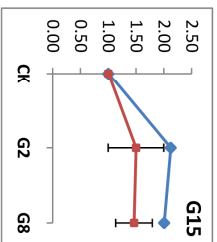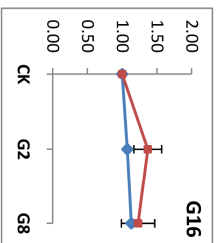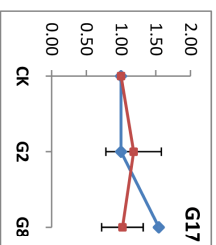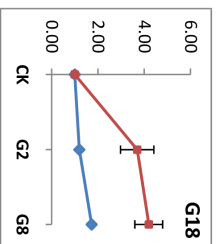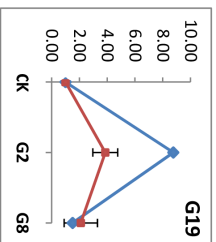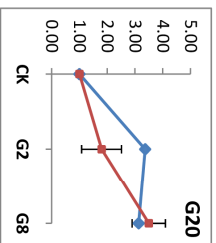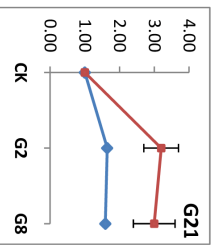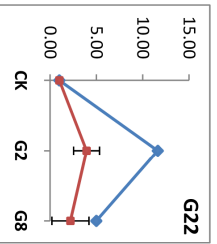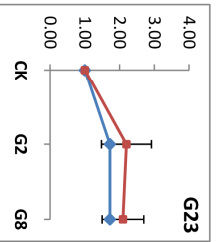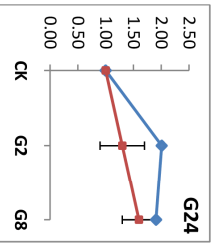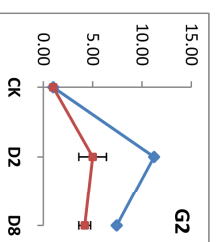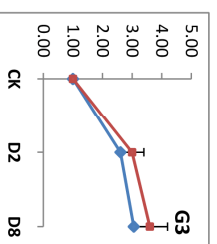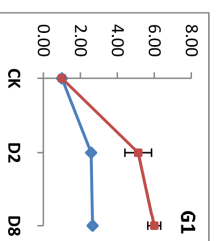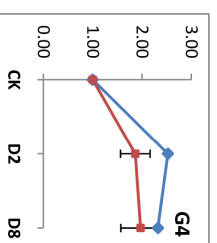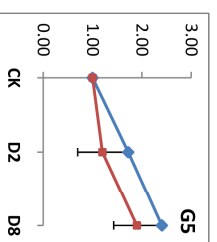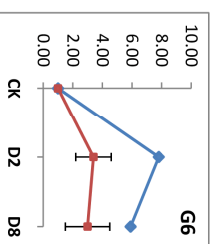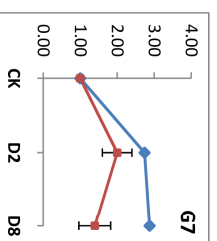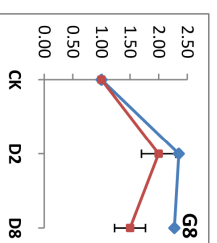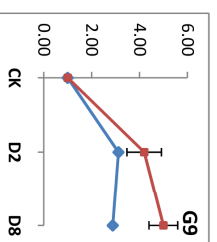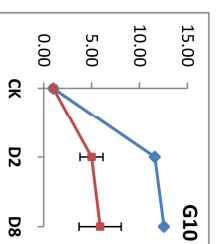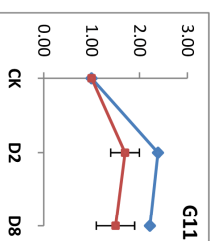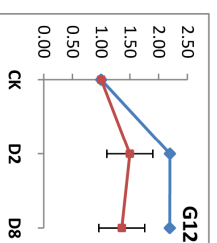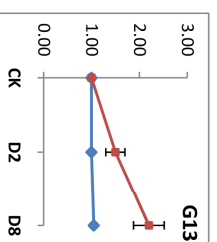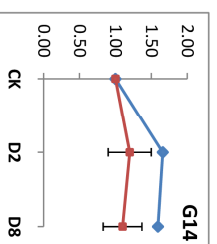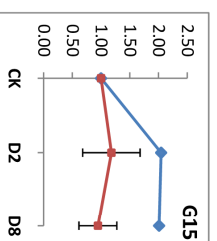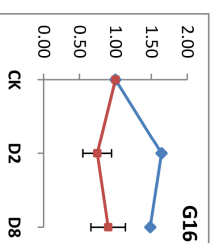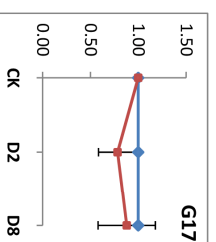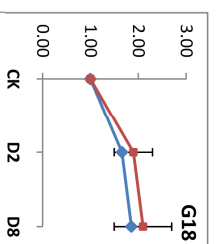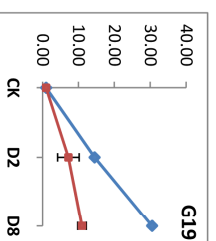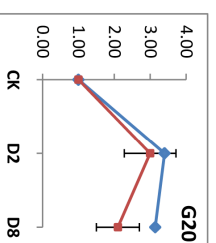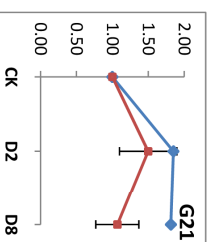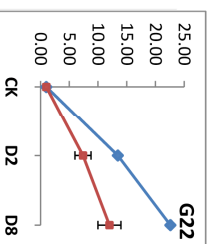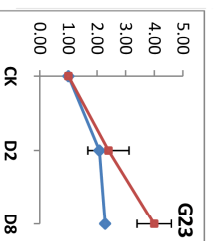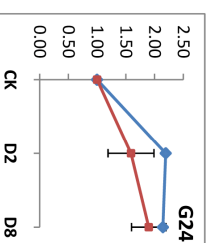

—◆— RNA-seq      —■— qPCR

Supplement: Figure S5 — X axis: D2, and D8 indicated expression levels at 2-hour and 8-hour under cold treatment (a), and G2 and G8 indicated expression levels at 2-hour and 8-hours under dehydarion treatment (b), respectively. Y axis indicated fold changes of expression levels, in which the expression level of CK was normalized to 1. Each PeTIFY gene was represented by gene ID starting with G as showing in Table S1. [file peerj-04-2620-s005.pdf]
