# Supplementary material for: Genome-wide identification and characterization of TIFY family genes in Moso Bamboo (Phyllostachys edulis) and expression profiling analysis under dehydration and cold stresses
Source: PeerJ. 2016 Oct 27;4:e2620. doi: 10.7717/peerj.2620 (PMC5088587; doi:10.7717/peerj.2620)
Supplement: Table S1 [file peerj-04-2620-s006.pdf]

Table S1 Primers used for qPCR analyses.

Gene ID			Sequence (5'->3')	Length	Tm
**PH01000008G2960**	**G1**	Forward primer	AGGCGGAACCGTCAGATTAC	20	59.82
		Reverse primer	GAGAAGTGGTCAAACACGGC	20	59.41
		Product length (bp)	142		
**PH01000038G0470**	**G2**	Forward primer	TGGGCAGAGTGCTAAGAATGG	21	60.07
		Reverse primer	TCACGATCGGGGCGATGC	18	62.59
		Product length (bp)	113		
**PH01000038G0510**	**G3**	Forward primer	CGTCAAGGCGGAGCAGAAGA	20	62.49
		Reverse primer	CCCGCCGTAGAAGATGGTCAG	21	62.55
		Product length (bp)	148		
**PH01000052G0540**	**G4**	Forward primer	GGAGAAGAAGGGTGGGCTAC	20	59.46
		Reverse primer	CGAGCCCTGACAACAAGTTC	20	59.13
		Product length (bp)	110		
**PH01000114G0660**	**G5**	Forward primer	GGGAGGTTGTCCCGATGGA	19	60.99
		Reverse primer	AACGACAGTGTGAGCGTGTT	20	60.46
		Product length (bp)	139		
**PH01000115G0020**	**G6**	Forward primer	TTGCTGCGGCAGTACATGAAG	21	61.54
		Reverse primer	GCCGCACCAACAACATCGG	19	62.61
		Product length (bp)	104		
**PH01000115G0040**	**G7**	Forward primer	GGCAAGAGCCGGAGGTTC	18	60.44
		Reverse primer	CGCGGCCGACATCTTCT	17	59.85
		Product length (bp)	78		
**PH01000158G0210**	**G8**	Forward primer	GGACGAACAGCTTCGCCAT	19	60.74
		Reverse primer	ATCCAAACTCCGCCACAGAT	20	59.38
		Product length (bp)	113		
**PH01000213G1380**	**G9**	Forward primer	GTGGTGAGCCTCCTGCC	17	59.68
		Reverse primer	CCGGCAGTTCTTGGGATGAC	20	61.03
		Product length (bp)	103		
**PH01000213G1410**	**G10**	Forward primer	AAGCCGGTGCGTCAAGG	17	60.34
		Reverse primer	CTTCTCTGACGTGGGGCAC	19	60.37
		Product length (bp)	122		
**PH01000310G0500**	**G11**	Forward primer	CTTCGGCGAAAACCGGGG	18	61.43
		Reverse primer	GGCGTCCTCGGAGCATC	17	59.93
		Product length (bp)	142		
**PH01000360G1030**	**G12**	Forward primer	ATGGAGATGTCTGCGTCCG	19	59.56
		Reverse primer	CCTCACCTTTGATCCCGAGG	20	59.82
		Product length (bp)	133		
**PH01000361G0580**	**G13**	Forward primer	AGGCAAGGGCGATCATATCC	20	59.67
		Reverse primer	GGTGGCGATCGTCATCTCTA	20	59.05
		Product length (bp)	75		
**PH01000549G0400**	**G14**	Forward primer	ATCATCTTCTACGACGGGCG	20	59.69
		Reverse primer	CATACCTTCCTCGCCTTCCC	20	59.89
		Product length (bp)	119		
**PH01000597G0660**	**G15**	Forward primer	GGAGGAGAAGACCCAGGAGT	20	59.96
		Reverse primer	AGCTGTGCAGGATTAGCTGG	20	60.11
		Product length (bp)	117		
**PH01000750G0690**	**G16**	Forward primer	GATGGCCACGATCCCCTG	18	59.89
		Reverse primer	GTCGAAGACGTAGACCTCGC	20	60.25
		Product length (bp)	132		
**PH01000836G0660**	**G17**	Forward primer	GTCGGGTCGAACACGCTC	18	60.8
		Reverse primer	TTTCCCGGAACCTCATCAGC	20	60.04
		Product length (bp)	121		

**PH01000878G0620**	**G18**	Forward primer	GTGAGCAGGATGGCGTCG	18	60.89
		Reverse primer	GAGCTGGTTGGACGAGACTG	20	60.39
		Product length (bp)	149		
**PH01001078G0280**	**G19**	Forward primer	ACGACCAGCAGCAGGTTTG	19	60.89
		Reverse primer	GATGCATCATGTGGTCGGC	19	59.35
		Product length (bp)	76		
**PH01001078G0420**	**G20**	Forward primer	TACATGCGGGAGCACCAAC	19	60.38
		Reverse primer	GAAGAGCTGCATGGTCCTGG	20	60.75
		Product length (bp)	120		
**PH01001584G0350**	**G21**	Forward primer	CGCTGGCCGAGGAGC	15	59.84
		Reverse primer	CCCTGGAACAGCAGAGTCAG	20	60.04
		Product length (bp)	82		
**PH01001852G0020**	**G22**	Forward primer	CTACAGACTCCACCACCCGA	20	60.61
		Reverse primer	GCGTTGGGTTCTTGTACGAC	20	59.49
		Product length (bp)	142		
**PH01002950G0020**	**G23**	Forward primer	CTCCCGTTCCCCCAATTCTC	20	60.11
		Reverse primer	TGTCAGCGCCGAAGTATCTC	20	59.9
		Product length (bp)	91		
**PH01144128G0010**	**G24**	Forward primer	ATTCGATAACGTCCCGGTGG	20	59.9
		Reverse primer	CGAATCTGATTTGCGGGCTG	20	59.97
		Product length (bp)	100		
**EF1**		Forward primer	GATGATTCCCACCAAGCCCA	20	60.03
		Reverse primer	TGGGTCCTTCTTCTCAACGC	20	59.97
		Product length (bp)	130		
