# Supplementary material for: Genome-wide identification and characterization of TIFY family genes in Moso Bamboo (Phyllostachys edulis) and expression profiling analysis under dehydration and cold stresses
Source: PeerJ. 2016 Oct 27;4:e2620. doi: 10.7717/peerj.2620 (PMC5088587; doi:10.7717/peerj.2620)
Supplement: Table S2 [file peerj-04-2620-s007.pdf]

Table S2 Comparison of Pfam analyses of two re-predicted gene models.

		Family	Description	Entry type	Clan	Start	End	Bit score	E-value
**PH01000361G0580**	Original	tify	tify domain	Domain	n/a	58	79	21.5	9.60E-05
	Re-predicted	tify	tify domain	Domain	n/a	58	88	37.3	1.1E-09
		CCT_2	Divergent CCT motif	Motif	CL0281	169	193	29.6	4.1E-07
**PH01000878G0620**	Original	tify	tify domain	Domain	n/a	80	113	49.3	1.9E-13
		CCT	CCT motif	Motif	CL0281	139	171	42.1	5.8E-11
	Re-predicted	tify	tify domain	Domain	n/a	80	113	45.5	3.1E-12
		CCT	CCT motif	Motif	CL0281	139	182	54.5	7.9E-15
		GATA	GATA zinc finger	Domain	CL0167	890	925	45.9	2.7E-12
		DUF3546	Domain of unknown function (DUF3546)	Family	n/a	1159	1261	51.6	8.5E-14
		ARS2	Arsenite-resistance protein 2	Family	n/a	1408	1620	115.5	3.4E-33
