# Supplementary material for: Genome-wide identification and characterization of TIFY family genes in Moso Bamboo (Phyllostachys edulis) and expression profiling analysis under dehydration and cold stresses
Source: PeerJ. 2016 Oct 27;4:e2620. doi: 10.7717/peerj.2620 (PMC5088587; doi:10.7717/peerj.2620)
Supplement: Table S3 [file peerj-04-2620-s008.pdf]

Table S3 Detailed protein annotation of re-predicted PH01000878G0620 by Pfam and NCBI.

Name	Accession	Description	Interval		E-value
TIFY	PF06200.10	TIFY domain	80	114	3.1E-12
CCT	PF06203.10	CCT motif	139	183	2.18E-18
UBN2_3	pfam14244	gag-polypeptide of LTR copia-type	282	381	7.74E-06
RVT_2	pfam07727	Reverse transcriptase (RNA-dependent DNA polymerase); It is usually indicative of a mobile element such as a retrotransposon or retrovirus.	758	795	1.69E-03
RNase_HI_RT_Ty1	cd09272	Ty1/Copia family of RNase HI in long-term repeat retroelements	857	888	4.05E-03
ZnF_GATA	PF00320.23	GATA zinc finger	889	924	2.70E-12
DUF3546	pfam12066	Domain of unknown function (DUF3546)	1157	1253	4.34E-32
ARS2	pfam07727	Arsenite-resistance protein 2	1408	1620	3.40E-33
