# Supplementary material for: Genome-wide identification and characterization of TIFY family genes in Moso Bamboo (Phyllostachys edulis) and expression profiling analysis under dehydration and cold stresses
Source: PeerJ. 2016 Oct 27;4:e2620. doi: 10.7717/peerj.2620 (PMC5088587; doi:10.7717/peerj.2620)
Supplement: Table S4 [file peerj-04-2620-s009.pdf]

Table S4 Paralogous and orthologous relationship among PeTIFY, OsTIFY, and, BdTIFY

Paralogues		Otholgoues			
		OsTIFY		BdTIFY	
PH01000038G0470	PH01000115G0020	LOC_Os03G08320.1		Bradi1G72600	
PH01000038G0510	PH01000115G0040	LOC_Os03G08330.1		Bradi1G72590	
PH01000114G0660	PH01000836G0660	LOC Os02g05510.1	LOC_Os06G48534.1	Bradi1G33980	
PH01000213G1380	PH01001078G0420	LOC_Os10G25290.1			
PH01000008G2960	PH01144128G0010	LOC_Os08G33160.1		Bradi3G36380	
PH01000360G1030	PH01000158G0210	LOC_Os07G42370.1		Bradi3G10820	Bradi1G21490
PH01001584G0350		LOC_Os03G47970.1		Bradi1G12330	
PH01001852G0020		LOC_Os03G08310.1		Bradi1G72610	
PH01000750G0690		LOC_Os03G52450.1		Bradi1G09550	
PH01000310G0500		LOC_Os03G28940.1			
PH01000213G1410		LOC_Os10G25250.1			
PH01002950G0020		LOC Os09g23660.1		Bradi4G29830	
PH01000052G0540		LOC_Os09G26780.1		Bradi4G31240	
PH01000597G0660		LOC_Os04G55920.1		Bradi5G24410	
