# Supplementary material for: Genome-wide identification and characterization of TIFY family genes in Moso Bamboo (Phyllostachys edulis) and expression profiling analysis under dehydration and cold stresses
Source: PeerJ. 2016 Oct 27;4:e2620. doi: 10.7717/peerj.2620 (PMC5088587; doi:10.7717/peerj.2620)
Supplement: Table S5 — Note: Expression level was present as RPKM value and log2 Normalized.Dehy indicates Dehydration. [file peerj-04-2620-s010.pdf]

Table S5 Expression levels of PeTIFY genes under dehydration and cold stresses.

GeneName	subfamily	Expression level									
		CK1	Dehy_2h-1	Dehy_8h-1	Cold_2h-1	Cold_8h-1	CK2	Dehy_2h-2	Dehy_8h-2	Cold_2h-2	Cold_8h-2
PH01001078G028	JAZ	0.00	4.21	5.48	2.84	0.25	0.00	3.51	4.38	3.42	0.93
PH01001852G002	JAZ	0.00	4.06	4.96	3.66	2.00	0.00	3.44	4.04	3.42	2.64
PH01000213G141	JAZ	1.35	4.74	5.68	3.78	3.02	1.37	4.88	4.26	3.99	2.20
PH01000038G047	JAZ	1.53	5.74	5.91	4.76	5.50	1.57	5.07	3.06	5.19	4.74
PH01000115G002	JAZ	1.83	6.06	5.97	4.06	5.79	2.01	5.33	3.86	4.48	4.70
PH01144128G001	JAZ	3.47	3.48	4.30	2.99	2.82	3.51	3.92	4.03	2.50	2.67
PH01000213G138	JAZ	3.57	6.21	6.56	5.54	6.59	3.84	5.96	4.77	5.85	6.50
PH01001078G042	JAZ	3.60	6.54	6.48	6.34	6.27	3.67	6.28	5.51	6.38	5.70
PH01000052G054	JAZ	3.82	5.26	5.57	4.58	4.47	3.88	5.04	3.79	4.67	3.30
PH01000158G021	JAZ	4.44	5.68	5.66	5.26	5.61	4.46	5.30	4.89	5.44	4.52
PH01000038G051	JAZ	4.49	6.20	7.93	5.53	5.71	4.76	6.60	6.99	5.77	5.69
PH01000115G004	JAZ	4.87	6.84	8.05	6.13	6.35	4.96	7.47	6.94	6.73	6.22
PH01000008G296	JAZ	5.21	7.28	7.75	6.26	6.22	5.19	6.98	6.97	6.32	6.00
PH01000360G103	JAZ	6.04	6.92	7.23	6.43	6.41	6.02	6.77	6.43	6.69	5.52
PH01000310G050	JAZ	6.35	8.05	8.13	9.03	8.62	6.29	7.83	6.41	9.10	8.29
PH01002950G002	JAZ	6.67	7.56	7.74	6.66	6.36	6.64	7.55	6.93	6.74	6.04
PH01000597G066	JAZ	7.13	7.50	7.48	7.61	7.22	7.11	7.21	6.87	7.88	7.09
PH01000361G058	JAZ	0.00	0.00	0.13	0.00	0.00	0.00	0.00	0.00	0.33	0.00
PH01000549G040	TIFY	4.39	2.80	2.86	2.23	1.05	4.35	3.59	3.00	2.33	0.02
PH01000836G066	ZML	0.00	0.00	0.00	0.00	1.25	0.00	0.00	0.00	0.00	0.00
PH01000114G066	ZML	1.01	1.05	1.35	0.67	2.03	1.37	0.82	1.67	0.54	1.16
PH01000878G062	ZML	1.85	1.37	1.63	0.02	1.84	1.81	1.30	1.63	0.92	1.05
PH01000750G069	ZML	2.52	1.70	1.48	0.02	0.32	2.32	1.76	1.27	0.47	0.55
PH01001584G035	ZML	3.45	3.01	3.33	2.23	2.61	3.68	3.32	2.80	2.88	2.17

Note: Expression level was present as RPKM value and log2 Normalized.Dehy indicates Dehydration.
