# Supplementary material for: Genome-wide identification and characterization of TIFY family genes in Moso Bamboo (Phyllostachys edulis) and expression profiling analysis under dehydration and cold stresses
Source: PeerJ. 2016 Oct 27;4:e2620. doi: 10.7717/peerj.2620 (PMC5088587; doi:10.7717/peerj.2620)
Supplement: Figure S1 [file peerj-04-2620-s001.pdf]

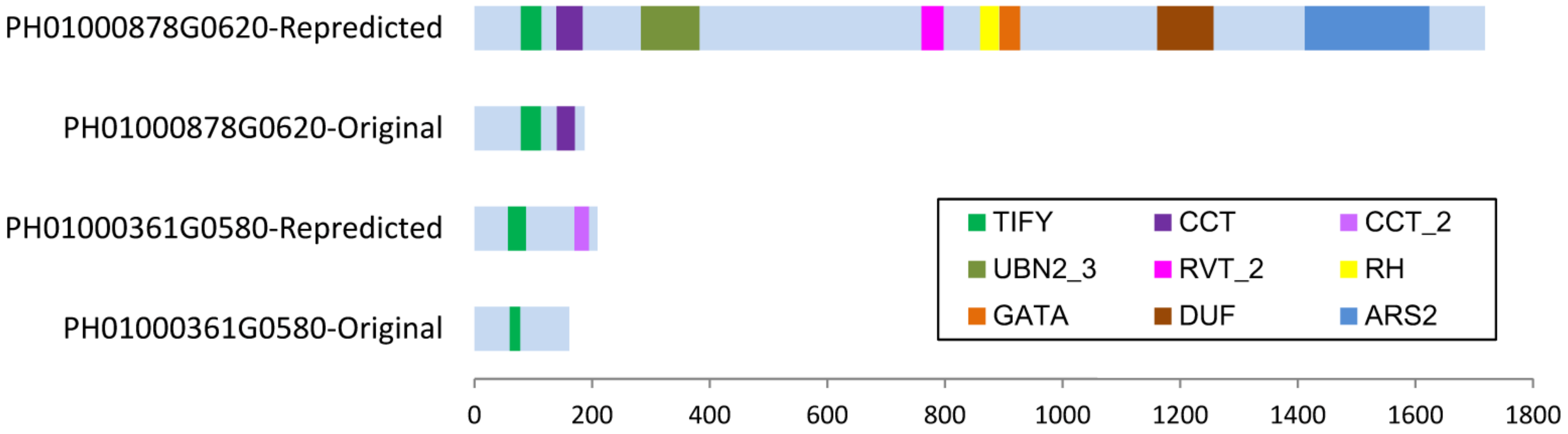
PH01000878G0620-Repredicted
PH01000878G0620-Original
PH01000361G0580-Repredicted
PH01000361G0580-Original
TIFY
CCT
CCT_2
UBN2_3
RVT_2
RH
GATA
DUF
ARS2
0
200
400
600
800
1000
1200
1400
1600
1800
